# Chronic Diffuse Pain and Functional Gastrointestinal Disorders After Traumatic Stress: Pathophysiology Through a Polyvagal Perspective

**DOI:** 10.3389/fmed.2018.00145

**Published:** 2018-05-31

**Authors:** Jacek Kolacz, Stephen W. Porges

**Affiliations:** ^1^Traumatic Stress Research Consortium, Kinsey Institute, Indiana University, Bloomington, IN, United States; ^2^Department of Psychiatry, University of North Carolina, Chapel Hill, NC, United States

**Keywords:** trauma, polyvagal theory, irritable bowel syndrome, functional gastrointestinal disorders, chronic pain, fibromyalgia, heart rate variability, respiratory sinus arrhythmia

## Abstract

Chronic diffuse pain disorders, such as fibromyalgia, and functional gastrointestinal disorders (FGIDs), such as irritable bowel syndrome, place substantial burden on those affected and on the medical system. Despite their sizable impact, their pathophysiology is poorly understood. In contrast to an approach that focuses on the correlation between heart rate variability (HRV) and a specific organ or symptom, we propose that a bio-evolutionary threat-related autonomic response—as outlined in the Polyvagal Theory—may serve as a plausible explanation of how HRV, particularly respiratory sinus arrhythmia (RSA), would index the pathophysiology of these disorders. Evidence comes from: (1) the well-documented atypical autonomic regulation of the heart common to fibromyalgia and irritable bowel syndrome reflected in dampened RSA, (2) the neural architecture that integrates the heart, pain pathways, and the gastrointestinal tract, (3) the common physical co-morbidities shared by chronic diffuse pain and FGIDs, many of which are functionally regulated by the autonomic nervous system, (4) the elevated risk of chronic diffuse pain and FGIDs following traumatic stress or abuse, (5) and the elevated risk of chronic diffuse pain and FGIDs in individuals with anxiety and panic disorders. This novel conceptualization points to a pathogenesis rooted in changes to brain-body autonomic feedback loops in response to evolutionarily-salient threat cues, providing an integrated biopsychosocial model of chronic diffuse pain and FGIDs and suggesting new, non-pharmacological treatment strategies.

## Introduction

Medically unexplained somatic problems including chronic diffuse pain and functional gastrointestinal disorders (FGIDs) are persistent, disabling, costly, and seen across all medical settings (1–3). However, their pathophysiology is poorly understood. Increasingly, there is a growing awareness that chronic diffuse pain and FGIDs are highly prevalent among those with a history of trauma or abuse (4, 5). Rates of abuse history tend to be highest among patients in pain and gastroenterology clinics (6) and one study found that of all females referred to a gastroenterology clinic, 67% had experienced sexual or physical abuse (7).

Despite this awareness, a pathophysiological mechanism that links trauma and abuse history to chronic pain and FGIDs is lacking. In this review, we use the framework provided by the Polyvagal Theory, an evolutionary neurophysiological model of the autonomic response to safety and threat (8–11). Within this framework, we outline a pathophysiological mechanism rooted in chronic autonomic threat responses that give rise to systemic changes in the regulation of pain pathways and the gastrointestinal tract. We then apply this framework to converging evidence from medicine, psychiatry, physiology, and neuroscience to provide a plausible model for the origins of post-traumatic chronic diffuse pain and functional gastrointestinal disorders in the absence of organic medical cause. While the pathogenesis described here may apply to a range of functional somatic disorders, this review focuses on fibromyalgia (FM) and irritable bowel syndrome (IBS) due to the wide clinical prevalence and abundant research related to these disorders.

Fibromyalgia is a chronic widespread pain condition impacting muscles, ligaments, and tendons. With a prevalence around 2–3%, it is among the most common types of chronic diffuse pain (12). It is estimated that women are about twice as likely to develop the disorder than men (13). A diagnosis is typically made based on the number of tender points in characteristic locations, chronicity, and exclusion of other disorders that may be causing pain. While the etiology of fibromyalgia is unknown and pathophysiology is poorly understood, there is growing understanding that emotional or physical trauma may trigger or aggravate symptoms (13).

Irritable bowel syndrome (IBS) is a gastrointestinal disorder characterized by chronic abdominal pain and altered bowel habits—diarrhea, constipation, or alternating episodes of both—in the absence of clear anatomical or physiologic abnormalities. The most recent criteria defines irritable bowel syndrome as recurrent abdominal pain, on average, at least 1 day per week in the 3 months before diagnosis that is associated with two more of the following: (a) relation to defecation, (b) association with change in the frequency of stool, (c) association with a change in the form (appearance) of stool (Rome IV criteria) (14, 15). It is the most frequently diagnosed gastro-intestinal condition, accounting for about 30% of all referrals to gastroenterologists (16) and is highly co-morbid with fibromyalgia (17). Based on a meta-analysis of 80 studies involving over 250,000 total subjects, the world-wide prevalence is estimated to be about 11.2% with higher rates in women than men (18). Like fibromyalgia, the etiology and pathophysiology of irritable bowel syndrome remain uncertain although emotional stress intensifies symptoms and hinders positive treatment outcomes (17).

We review evidence for fibromyalgia and irritable bowel syndrome as arising from a chronic autonomic response that may be triggered or exacerbated by evolutionary bio-behavioral processes that regulate the brain and body in response to threat. In pursuit of this, this paper reviews: (1) the elevated prevalence of fibromyalgia and irritable bowel syndrome among sexual abuse and rape survivors, (2) the organizing autonomic threat-response principles drawn from the guiding framework of the Polyvagal Theory that explain the adaptive function of shifts in autonomic state reflected in respiratory sinus arrhythmia (high frequency heart rate variability), (3) the autonomic and neurophysiological systems that regulate pain and gastrointestinal function, (4) empirical evidence of diminished RSA reflecting altered autonomic function in fibromyalgia and irritable bowel syndrome patients, (5) common autonomically-regulated co-morbidities in fibromyalgia and irritable bowel syndrome patients, and (6) evidence for autonomic disruption after trauma. The understanding of fibromyalgia and irritable bowel syndrome as systemic dysfunction produced by a chronic autonomic threat response provides novel opportunities for treatment that targets the brain-body system, including the brain-gut axis, rather than treating individual symptoms.

## Fibromyalgia and irritable bowel syndrome after sexual abuse and rape

Sexual and physical abuse in both childhood and adulthood are a widespread problem. Based on a series of large-scale meta-analyses, international prevalence rates are estimated to be 13% for childhood sexual abuse (8% males, 18% females) and 23% for childhood physical abuse (19). In the US, the Centers for Disease Control and Prevention estimate that the lifetime prevalence of rape is 19% in women and 2% in men and intimate partner violence prevalence is 32% in women and 28% in men (with *severe* intimate partner violence prevalence at 22 and 14%, respectively) (20). Notably, these higher rates of sexual and physical abuse among women are paralleled in the gender-specific prevalence of fibromyalgia and irritable bowel syndrome. Women are about twice as likely to have a fibromyalgia diagnosis (13) and have 1.67 greater odds of having irritable bowel syndrome than men (18).

Converging evidence across many studies shows that abuse history is a strong predictor of fibromyalgia. In a meta-analysis of 18 studies with a total of over 13,000 combined participants, Häuser and colleagues found that sexual and physical abuse in both childhood and adulthood predicted greater odds of fibromyalgia (odds ratio point estimate range: 1.94–3.07) (21). In their meta-analysis, Paras and colleagues found that rape survivors have especially high odds of fibromyalgia diagnosis (*OR* = 3.27) (22). These risk factors are higher in fibromyalgia patients than those with rheumatoid arthritis, highlighting the specificity of the role of abuse and trauma in FM pathogenesis compared to a pain disorder with a known organic cause (23).

Rates of gastrointestinal problems such as irritable bowel syndrome are similarly elevated in survivors of abuse. Meta-analytic evidence supports this with childhood sexual abuse being associated with higher risk of gastrointestinal problems (24). It is estimated that individuals with a history of sexual abuse are about twice as likely to develop abdominal pain and gastrointestinal problems than those without an abuse history (25). As seen with fibromyalgia, rape survivors are among those with the highest risk, with meta-analytic methods suggesting their odds of having a functional gastrointestinal disorder are about four times greater than those without an abuse history (22). Those with an abuse history also have higher severity and quantity of GI symptoms and seek medical help more often (26).

Overall, these robust associations suggest that abuse and trauma experiences may be key to understanding the pathogenesis of fibromyalgia and irritable bowel syndrome. However, a comprehensive integrative model to explain the mechanisms through which traumatic experiences affect these chronic disorders is lacking.

## The polyvagal theory

The brain-body connection is composed of integrated sensory, interoceptive, and regulatory systems that monitor internal body conditions and the external environment, coordinating homeostatic processes required for maintenance of biological functions as well as responses to threats from inside and out (27–30). Within the body, interoceptive signals continuously provide homeostatic information about the body's physiological condition to the brain. In complement, exteroception (evaluation of external conditions) provides information about environmental challenges and threats, to allow for the flexible shifting of resource allocation to meet external demands. Detection of both internal and external threats or perturbations can disrupt homeostatic processes in the interest of allocating resources for addressing the threat.

The Polyvagal Theory (8–11) provides a neurophysiological framework for understanding the evolutionary history of mammalian nervous system and its relation to the anatomic and functional organization of the human brain-body connection. Over the course of evolution, vertebrate nervous systems have elaborated on and appended to the earliest and most primitive of these brain-body systems. With time, newer systems increased precision, speed, and complexity, as well as evolved new functions that respond to novel environmental and social needs. These newer neurophysiological systems did not replace older systems, but instead became integrated with older functions.

Some of the first of these regulatory systems, observed in the earliest vertebrates, were powered by hormones and facilitated relatively slow, diffuse reactions in the brain and body. However, with time, the evolution of the autonomic system afforded more rapid and targeted control. With the heart serving as a key metabolic structure, the earliest neural regulatory pathways evolved to provide control over its function. These efferent pathways emerged from an area of the brainstem known as the dorsal motor nucleus of the vagus (DMX), adjacent to the target site of the sensory afferent pathways that provides information from the heart and target organs. Together, this coordinated efferent-afferent system formed the dorsal vagal complex (DVC). This regulatory circuit was well developed in early vertebrate evolution and emerged in primitive vertebrates such as cartilaginous fish, integrating information arising from the body and higher brain structures. In humans, this system has few fibers innervating the heart, with most efferent projections innervating organs below the diaphragm, including the gastro-intestinal tract, to regulate digestive and metabolic functions.

The emergence of a spinal sympathetic nervous system (SNS) in bony fish, with its broad scope and target specificity, provided a rapid and coordinated fight/flight system for mobilizing the body in response to threats. This system built on the architecture of the DVC, utilizing information traveling from the body and exteroceptive higher brain regions to coordinate threat-related response needs. In humans, this system regulates a wide range of tissues and structures including the digestive tract, heart, and lungs.

The phylogenetically newest autonomic system is the ventral vagal complex (VVC), which emerged during the transition from primitive extinct reptiles to mammals. Like the SNS before it, this system built on the integration of afferent information arising from the body and descending signals from higher brain structures to respond to internal and external needs. Its efferent arm emerged as cell bodies of vagal efferent source nuclei of the DMX migrated ventrally, forming a second and distinct cardioinhibitory nucleus known as the nucleus ambiguus (NA) and became integrated with circuits that regulate the bronchi and muscles of the face and head. Unlike earlier systems, which promoted defense-related survival responses, this new autonomic face-heart connection formed in concert with the mammalian dependence on social-affiliative behaviors. It provided a substrate for communication of defense or affiliative states via vocalization acoustic features (via regulation of the larynx and pharynx) (31, 32) and facial expressions, as well as providing capacity to dampen older survival-based response systems.

In mammals, all three systems contribute to maintaining homeostatic function and coordinating responses to internal and external perturbations. Information from within is integrated in signals arising from afferent pathways and converging on brainstem structures, most prominently the nucleus tractus solitarius (NTS), while top down signals from higher brain regions, including sensory systems, provide information about external threats. This integrated interoceptive and exteroceptive information is projected to higher-level brain structures and to visceral organs via the source nuclei of the motor pathways involved in the DVC, SNS, and VVC to coordinate responses. This integrated information also interacts with neuroception, the subconscious assessment of threat and safety in the environment (named in contrast to consciously experienced sensory perception) (33, 34). Importantly, the non-conscious threat detection that occurs in both the body and higher-level brain centers suggests that individuals may not be aware of threat-response triggers. However, they may consciously experience the threat-related changes in brain and body regulation (e.g., urge to defecate, feelings of anxiety).

The Polyvagal Theory proposes that the evolution and individual anatomical pathways of the DVC, SNS, and VVC give rise to an ordered response hierarchy that promotes coordinated responses to threats. Under normal homeostatic conditions, each system is involved in basal functions for organism maintenance. However, under threat conditions, the function of individual systems can be recruited to regulate metabolic resources as needed. Phylogenetically newer systems are primary responders; older systems are recruited as threat persists. This phylogenetically ordered response hierarchy is consistent with the Jacksonian principle of dissolution (35). First, under threat, the VVC, the most rapidly responsive system, withdraws its inhibitory influence on mobilization. Since a component of HRV, known as respiratory sinus arrhythmia (RSA; see below), is primarily a product of this circuit, it is often used as an index of the strength of this circuit. Second, the SNS can further ramp up mobilization and promote fight/flight behaviors. Finally, when recruited for defense responses, the DVC produces an efferent vagal surge that inhibits metabolic functions by slowing heart rate, reducing digestive processes, and promoting behavioral shut down. Notably, all three autonomic systems feature cardio-regulatory pathways; modulating cardiac function is key to altering metabolic resources for body responses. Calm, affiliative social engagement requires dampening of threat-related bodily mobilization (this mobilization brake must be removed for efficient body mobilization), fight/flight mobilization requires ratcheting up available resources, and behavioral shut down requires reduction of metabolic function.

Though acute neural threat reactions are adaptive and necessary, chronic state shifts that maintain threat responses can be a risk factor for body dysfunction and disease. The brain-body connection is composed of multiple integrated feedback loops and the chronic maintenance of threat responses can lead to a “compromised” functional state. Chronic compromised states may give rise to the emergence of functional gastrointestinal disorders and altered pain signaling as part of a chronic systemic, rather than an event related organ- specific, pathophysiology. These changes in homeostatic functions may be self-maintaining or cascading even after the threat has been lifted due to long-term alterations in set points, learned responses to threat cues, and the symptoms themselves propagating threat-reaction.

## Neural circuits involved in pain and gastrointestinal regulation

### Neural pain regulation in the periphery

The detection and appropriate responses to noxious or dangerous stimuli from without and within relies partly on the propagation of pain signals through the nervous system (nociception). Mechanisms integrating nociceptive sensory signaling, spinal pathways, and central regulation allow dynamic flexibility for acute and long-term safety- and danger-related functions. Pain signal regulation is a normal part of a nervous system defense response, such as the body's illness reaction that activates and sensitizes afferent nociceptive neurons (36). This adaptive pain signal modulation, which relies on integrated afferent-efferent brain-body feedback loops may be compromised in chronic-long term states of threat response.

Spinal nociceptive pathways are not passive conveyors of information from the body to brain, but rather under dynamic endogenous regulatory control that includes mechanisms contributing to sensitization and inhibition. During typical homeostatic states, active inhibition of pain signals modulates the strength of nociceptive response levels, silences nociceptive neurons in the absence of noxious stimuli, and impedes the spread of excitatory signals between sensory modalities and somatotopic borders (37). These inhibitory signals are prepotent, typically tonically active, crucial for constraining spinal nociceptive signaling, and have a large portion of the spinal neural architecture dedicated to them (38, 39). Dampening of these inhibitory mechanisms or facilitation of pain-related signaling can lead to hyperalgesia, allodynia, or spontaneous pain (37, 39). Somatic and interoceptive systems provide constant dynamic input from the body to the brain, and pain signaling from these system is highly integrated with homeostatic threat-responsive systems (40). Changes in homeostatic and threat-response feedback loops thus may interact with these incoming afferent signals, altering efferent (motor) output and perceptual qualities.

The sensitization and inhibition of spinal pain pathways is under the influence of brain-body feedback loops. These include multiple brain areas related to survival and threat related functions as well as the sympatho-adrenal system and vagal afferent pathways in the periphery. Descending modulation from the brain includes the periaqueductal gray, the rostroventral medulla, the lateral and caudal dorsal reticular nucleus, and the ventrolateral medulla (39, 41). Notably, several of the brainstem areas that are involved in pain modulation–including the medullary raphe and ventromedial reticular region-are also involved in the regulation of autonomic sexual functions and defense behavior (42), providing the substrate for functional coupling of survival- and threat-related states with pain.

Peripheral mechanisms that include vagal and spinal pathways are also involved in regulating nociception. Under normal homeostatic conditions, active nociceptive inhibitory control is maintained by spontaneous tonic vagal afferent activity (36, 43). Threat-related subdiaphragmatic vagal afferent signaling or disruption of tonic activity to the brain can trigger defense reactions that affect pain not just in the viscera but in the superficial and deep somatic tissues as well (36, 44). These signals can activate second-order NTS neurons, leading to an illness response cascade that facilitates nonciceptive impulse transmission in the spinal cord, inhibition of digestion, and biobehavioral state changes including immobility and sleep increase (36). In rodents, the nociceptive sensitivity induced by severing vagal afferents shows an extended time delay, with maximum sensitization of the paw-withdrawal threshold being reached by about 1–3 weeks post-surgery and involving interactions with the sympatho-adrenal system (45). In this study, this level sensitization endured over the course of the study, which lasted 8 weeks, indicating that the sensitization was chronic. In light of the present model, vagal afferent severance may mimic an extreme loss of vagal afferent flow and feedback loop, promoting a state of heightened autonomic threat response. This slow time frame to reach maximum sensitization may reflect complex, slow acting processes that give rise to latent sensitization emerging some time after the initial traumatic insult.

### Neural gastrointestinal regulation

The digestive tract is innervated by a rich complex of afferent, efferent, and inter-neurons. Most of these neurons form the enteric nervous system, the intrinsic gastrointestinal network containing approximately an equal number of neurons to the spinal cord (47, 48). The enteric nervous system produces local reflex loops that coordinate gastrointestinal functions. The neural brain-gut connection is formed by spinal and vagal afferent pathways and projections from the DVC and SNS. Visceral afferent vagal and spinal projections carry chemical, mechanical, and, inflammatory, immune, toxicity, and noxious information. DVC and SNS efferent projections synapse mostly with the neurons of the enteric system, rather than the digestive tract itself (though several sites are directly innervated, such as those mediating defecation by the DVC and vasoconstriction by the SNS).

For the most part, under normal conditions, basic digestive functions are delegated to intrinsic enteric processes (though spinal and vagal pathways are employed for reflexes to coordinate GI function across wide or multiple regions) (46, 47). However, under conditions of internal and external threat, SNS and DVC pathways can override typical gastrointestinal local reflexes as part of coordinated organism responses (48, 49). In such situations, digestion can be inhibited or bowels can be cleared to facilitate more energy for other metabolic needs. These functions are executed by alterations to digestive secretions, motility, and—via the DVC—defecation. Sympathetic outflow to the gut is inhibitory, slowing transit and diminishing secretions. The DVC includes both excitatory and inhibitory functions. Gastrointestinal function changes induced by DMX stimulation in animals co-occur with heart rate and respiration slowing (50), consistent with the view that in contrast to the fight-flight functions of the SNS, recruitment of the DVC during challenge may promote bio-behavioral shut down.

These gastrointestinal function circuits are coordinated with afferent information arising from the body to the NTS (51) as well as the function of the VVC. Although the VVC does not directly innervate the gut, the stimulation of its efferent source nuclei (the NA) in rodents inhibits digestive function via feedback through the subdiaphragmatic vagal pathways that include the DVC (52). This suggests that monitoring of VVC function, via indices like RSA, can provide an indirect window into the state-related modulation of GI function.

## Fibromyalgia and irritable bowel syndrome: manifestations in vagal regulation of the heart

### Respiratory sinus arrhythmia (RSA): an index of ventral vagal complex activity

Multiple neural mechanisms influence the heart's internal pacemaker. While heart rate is a global measure of metabolic state, understanding the neural mechanisms mediating dynamic changes in cardiac output provides information for metrics capable of indexing the influence of vagal pathways on the heart. Although the intrinsic firing rate of the sino-atrial node, the heart's pacemaker, may be relatively fixed, the heart does not beat at a constant rate in healthy individuals. The heart beat is modulated by the transitory inhibition of the pacemaker by vagal pathways. When the spontaneous respiration rate is manifested in the heart rate pattern it is called respiratory sinus arrhythmia (RSA).

References to RSA were made in the early twentieth century. Wundt observed that “respiratory movements are … regularly accompanied by fluctuations of the pulse, whose rapidity increases in inspiration and decreases in expiration.” (53) Hering described the functional relation between the amplitude of RSA and cardiac vagal tone, stating “It is known with breathing that a demonstrable lowering of heart rate is indicative of the function of the vagi” (54) (see (55, 56) for historical perspectives on heart rate variability and the autonomic nervous system). Since the neural mechanisms mediating RSA are well understood as the functional output of myelinated efferent vagal pathways, we focus on RSA and not on other metrics of heart rate variability, the origins of which have yet to be clearly defined.

Contemporary neurophysiology has supported these early reports and provided increased detail on their mechanisms. Humans and non-human mammals have cardioinhibitory neurons housed within the nucleus ambiguus of the VVC (55). These cardioinhibitory neurons receive excitatory input from the NTS (facilitating heart beat slowing) and inhibitory influences from brainstem reflexes linked to the respiratory rhythm (57, 58). This process gives rise to heart rate slowing during exhalation, mediated via vagal efferent outflow from the nucleus ambiguus. Thus, respiratory sinus arrhythmia, heart rate variability in the frequency of spontaneous respiration (also termed high frequency heart rate variability), provides an index of nucleus ambiguus cardioinhibitory function that separates its effect from that of other cardio-regulatory influences that lack a respiratory rhythm.

As described in the Polyvagal Theory, the mammalian nucleus ambiguus is a component of the VVC that is involved in facilitating affiliative social behavior and dampening threat-related reactivity (see above). It integrates the regulation of the body with safety- and threat-related states, forming an afferent-efferent system. Changes in signaling within the NTS (an area receiving information from the body vagal and spinal afferents) alter functional output of cardioinhibitory neurons in the nucleus ambiguous (58) and gastrointestinal regulation via the DMX (51). If the outflow of the VVC system is dampened or withdrawn (giving rise to low RSA), principles of dissolution would predict that more primitive threat-reactivity systems would be activated, reflecting a threat-response bodily profile marked by poorer homeostatic digestive function and potentially sensitized pain signaling.

### Evidence for dampened RSA in fibromyalgia and irritable bowel syndrome

In accordance with this prediction, several reviews and meta-analytic analyses document that fibromyalgia is associated with depressed heart rate variability including RSA, which is often reported as high frequency HRV (59–61). Furthermore, there is mounting evidence that interventions that decrease pain in fibromyalgia also result in increased HRV. A recent study reported that individuals whose fibromyalgia symptoms benefited from resistance training also responded with an increase in HRV (62), while an intervention that used breathing biofeedback documented that practice increased participant RSA and decreased pain (63).

Similarly, irritable bowel syndrome has also been linked to reduced VVC control of the heart as indexed by RSA. A meta-analysis based on 7 studies found that RSA was depressed in irritable bowel syndrome patients compared to healthy controls (64). This dampened VVC control may be especially pronounced in the subtype of irritable bowel syndrome characterized by predominant constipation, severe abdominal pain, and a comorbidity of anxiety or depression (65).

That both fibromyalgia and irritable bowel syndrome are marked by low amplitude RSA supports the hypothesized role of dampened VVC control in these disorders and suggests these disorders are associated with a state of heightened threat-related autonomic state. This link is so consistently observed that low heart rate variability has even been proposed as a “biomarker” of fibromyalgia (61, 66). However, the lack of specificity of low HRV as unique to fibromyalgia and the functional relation between VVC function to broad safety- and threat-related functions warrants a more systemic autonomic interpretation of this link.

**Figure 1:**
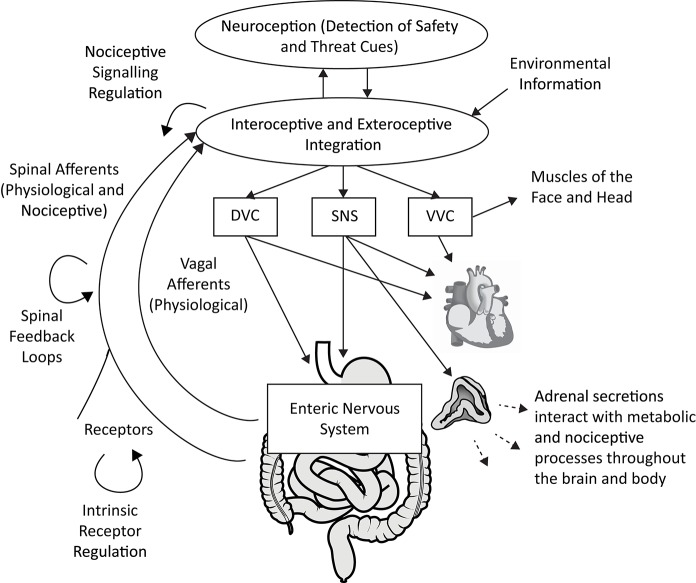
A polyvagal perspective on autonomic regulation of nociceptive signaling and gastrointestinal function. Interoceptive and exteroceptive information is constantly monitored and interacts with neuroception, the non-conscious process of detecting safety and threat cues. These signals are used to regulate adaptive biobehavioral responses to current conditions. Nociceptive signaling is regulated in concert with top-down processes involving the brainstem and higher brain structures, vagal afferent signaling, spinal feedback loops, and the sympatho-adrenal system. The gastrointestinal tract is regulated by the dorsal vagal complex (DVC) and sympathetic nervous system (SNS), partly via actions on the enteric nervous system. The heart can serve as an index of autonomic circuits, with respiratory sinus arrhythmia (high frequency heart rate variability) providing an index of ventral vagal complex (VVC) function. Acute and chronic threat responses can alter neural feedback loops. Anatomical graphics are drawn from Wikimedia Commons and used in accordance with the Creative Commons license. |Adrenal gland by DBCLS 
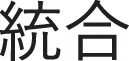
TV, CC BY 4.0, https://commons.wikimedia.org/w/index.php?curid=55201790 | Heart by Sheldahl, CC BY-SA 4.0, https://commons.wikimedia.org/w/index.php?curid=53328950 | Digestive tract by Olek Remesz (public domain), CC 2.5, https://commons.wikimedia.org/wiki/File:Tractus_intestinalis_esophagus.svg.

Polyvagal Theory proposes an alternative interpretation of the covariation of HRV with both fibromyalgia and irritable bowel syndrome. Consistent with the integrated model of the autonomic nervous system described in the theory, atypical heart rate variability is not interpreted as a biomarker of any specific disease. Rather, depressed respiratory sinus arrhythmia (high frequency heart rate variability) is proposed as a neurophysiological marker of a diffuse recalibration of the autonomic nervous system following an adaptive complex autonomic reaction to threat. We propose that an initially adaptive neural response to threat, via visceral afferent feedback from the visceral organs to the brainstem, may result in a chronic reorganization of autonomic regulation observed in vagal regulation of the heart (i.e., depressed RSA) in conjunction with altered subdiaphragmatic organ function and afferent pain signaling (see Figure 1).

## Fibromyalgia and irritable bowel syndrome co-morbidity and co-occurring autonomic symptoms

In addition to the shared VVC dampening in both fibromyalgia and irritable bowel syndrome, these disorders also share a high co-morbidity and overlap in autonomic symptoms. Fibromyalgia occurs in about 49% of irritable bowel syndrome cases (17). Both diagnoses are also associated with other autonomic and state-regulation problems. Irritable bowel syndrome patients have increased rates of non-digestive pelvic pain, chronic fatigue, sleep problems, syncope and dizziness (67), as well as heightened visceral and cutaneous pain perception (68). Fibromyalgia co-morbidities are similar and include chronic fatigue, sleep problems, elevated rates of visceral pain and sensory hypersensitivities (13). Furthermore, self-reported autonomic problems - including issues with subdiaphragmatic organs, orthostatic, vasomotor, and secretomotor functions - are elevated in fibromyalgia patients and overall severity of fibromyalgia symptoms is associated with severity of these problems (69).

Fibromyalgia and irritable bowel syndrome are both associated with elevated sexual and reproductive system problems that may be a manifestation of general autonomic function. Individuals with irritable bowel syndrome have elevated rates of vulvodynia, dysmenorrhea, amenorrhea, and irregular menstruation (67). Likewise, fibromyalgia is related to increased incidence of dysmenorrhea (13). In addition, women with fibromyalgia experience blunted sexual desire and arousal, fewer experiences of orgasm, and increased pain during intercourse (70).

The co-occurrence of fibromyalgia and irritable bowel syndrome with their overlapping autonomic co-morbidities suggest a role for a general systemic dysfunction as an underlying pathophysiology that gives rise to chronic diffuse pain and functional gastrointestinal problems. This points to the possibility that integrated brain-body reactions may produce long-term systemic changes that give rise to chronic disease and dysfunction related to evolutionary threat responses. Abuse or psychological trauma provides an especially potent experience that may re-tune the nervous system toward a chronic threat response.

## Traumatic stress is related to altered autonomic function

As reviewed above, the human autonomic nervous system is tuned to rapidly respond to a wide range of external and internal conditions. Many mild events that challenge the nervous system's resources are part of everyday life and trigger adaptive acute reactions. However, traumatic experiences may be especially potent in re-calibrating the autonomic nervous system toward a state that supports chronic defense responses. These long-term chronic changes, which affect autonomic regulation and the function of the brain and body, are observed across a range of traumatic events, including physical and sexual abuse in both childhood and adulthood.

These traumatic experiences may trigger a chronic threat-related brain-body response. A recent meta-analysis showed that RSA is dampened in individuals with Post-Traumatic Stress Disorder (PTSD) (71). This is consistent with a model of dampened VVC activity, as part of a chronic threat response, being implicated in long term effects following a trauma. Emerging evidence also suggests that a range of interventions including biofeedback and mindfulness that improve PTSD symptoms also increase heart rate variability (72–74). Importantly, however, VVC regulation may be dampened and trigger somatic problems even in the absence of a PTSD diagnosis. Although empirical research in this domain is currently scant, one study found that women with an abuse history have low RSA compared to health controls even when they do not meet diagnostic criteria for PTSD (75).

## Fibromyalgia and irritable bowel syndrome comorbidities with anxiety, panic disorders, depression, and somatic function

Anxiety disorders and depression are common sequelae following traumatic events. Both fibromyalgia and irritable bowel syndrome co-occur with panic disorder, generalized anxiety disorder, and PTSD (13, 76). It is estimated that 30–50% of fibromyalgia patients also have anxiety and/or depression at the time of diagnosis (12). A meta-analysis shows that across studies, depression symptoms are elevated in fibromyalgia and irritable bowel syndrome and anxiety is elevated in irritable bowel syndrome (77). Patients with GI dysfunctions, who have an abuse history, have higher rates of panic symptoms and depression as well as autonomically-related somatic symptoms such as sleep disturbance and pelvic pain than those without abuse history (26), suggesting a higher likelihood of systemic brain-body dysfunction.

## A new model of post-traumatic chronic pain and functional gastrointestinal disorders

The reviewed converging evidence, viewed through the lens of organizing principles derived from Polyvagal Theory, proposes a plausible pathway through which chronic subdiaphragmatic organ disease and pain may be based in the evolutionary neurophysiological defensive states elicited by abuse and life threat. These chronic systemic functional problems are reflected in regulation of the heart by the VVC (as measured by RSA), providing a portal for identifying disorders of visceral organ function. This novel model describes a possible mechanism through which trauma may lead to the pathophysiology of chronic pain and functional gastrointestinal disorders. When organic cause is not evident, functional GI problems or chronic diffuse pain may be a marker of a traumatized nervous system.

Although the link between sexual and physical abuse is well documented, there are many other experiences that may trigger chronic neurophysiological defense responses in chronic pain and FGIDs. Other traumas that trigger defense states, including invasive surgical procedures, may have similar effects. Many infants born prematurely require chronic intensive medical interventions to survive. These interventions, although enhancing survival, are marked by repeated pain-inducing procedures, surgery, medication, and maternal separation at a time when the regulation of the autonomic nervous system, particularly the VVC, is still developing. These experiences may all have the capacity to trigger a life-threat challenge to the nervous system, which may alter ANS function in the long term. Adolescents who were born premature have more tender points and lower tender thresholds than those born full term (78) and a recent study found that 62% of female fibromyalgia patients reported a gestation of <38 weeks (this gestational age cut off is based on the study sample median and provides preliminary evidence for the proposed model until a study with more clinically meaningful criteria is conducted) (79).

Our review has focused on the chronic pain disorder fibromyalgia, since it is among the most common types of chronic diffuse pain and has been widely researched. However, at least 10% of the general population has chronic diffuse pain that is not diagnosed as fibromyalgia, with little specific disease or obvious abnormality to explain symptoms (12). Notably, sexual abuse history is also associated with elevated rates of pelvic and nonspecific chronic pain (22). It is possible that these non-specific pain problems may have an underlying autonomic mechanism involving both vagal efferent and afferent pathways conveying information between the brainstem and subdiaphragmatic organs.

Similarly, our review of functional GI disorders has been limited to irritable bowel syndrome since this is the most widely reported and studied GI disorder. However, it is possible that the range of functional gastrointestinal disorders lacking obvious anatomical or physiologic causes that do not meet irritable bowel syndrome criteria—including functional constipation, diarrhea, abdominal bloating, and distension (15)—may have a pathophysiology rooted in chronic defense states. For instance, functional dyspepsia, a disorder marked by bloating, upper abdominal pain or discomfort, and indigestion in the absence of organic disease, may also be part of a chronic subdiaphragmatic immobilization or shut down response. Notably, the risk of functional dyspepsia is elevated among childhood sexual abuse and war survivors (80), co-occurs with elevated rates of depression and anxiety (77), is marked by a lack of the typical gastric antral motility reactivity to mental stress exhibited by healthy controls (81), is associated with decreased RSA (81, 82), and successful symptom treatment by acupuncture concurrently increases RSA (83).

## Experimental evidence for the proposed model and insights for the development of new treatment approaches

A conceptualization of pain and GI regulation as part of an integrated nervous system that is capable of dynamic state-related shifts provides an optimistic opportunity for new treatment targets. Rather than focusing on disease symptoms as organ, tissue, or pathway-specific, the understanding of the organizing principles through which the nervous system regulates visceral organs and afferent signals may lead to novel approaches that draw on evolutionary neurophysiological feedback loops. Treatments that target the autonomic nervous system, rather than the digestive system or a pain source, provide a direct test of the proposed model. With the rising availability of vagus nerve stimulation devices, there is increasing evidence that targeting ANS function can indeed improve symptoms.

Recent applications of electrical vagus nerve stimulation show promise for both treatment of pain and functional gastrointestinal disorders. These treatments stimulate afferent vagal signaling, rather than the GI tract or painful regions. This signaling may increase VVC regulation and strengthen the body's natural safety-related feedback loops that inhibit pain signaling and promote efficient GI function. A pilot study showed that an implanted vagal nerve stimulator had positive effects in a small sample of treatment-resistant fibromyalgia (84). This suggests that vagal nerve stimulation likely influences general noxious- and pain-related pathways and may have benefits for a range of neurophysiologically-linked problems. Promising results have been observed for respiratory-gated auricular vagal afferent nerve stimulation (RAVANS) in chronic pelvic pain patients, which include reductions in anxiety (85). Benefits have also been observed for GI function. In healthy participants, non-invasive transcutaneous electrical vagal nerve stimulation (t-VNS) with deep slow breathing increased both musculoskeletal pain threshold and gastroduodenal motility (86). The stimulation also increased heart rate variability, consistent with a model of VVC activation inhibiting pain pathways and stimulating efficient gastrointestinal functions. In addition, a randomized, sham-controlled double-blind study found that an ear-attached cranial nerve stimulator (which includes the vagus nerve as a target) improved GI pain symptoms in adolescents with pain-related FGIDs (87). These studies provide preliminary experimental evidence for the proposed model of ANS afferent signaling as a part of the pathophysiology of FGIDs and FM.

Beyond vagus nerve stimulation, a polyvagal approach also predicts that evolutionary cues that trigger neurophysiological safety responses and remove threat cues may likewise have treatment potential. Empirical work based on these evolutionary neurophysiological cues is needed. However, there is mounting evidence that mammalian safety-cueing acoustic features are embedded in frequency bands and spectro-temporal modulation (31, 32) and preliminary work demonstrates that these properties may be effective in promoting VVC activation for clinical applications (88). Other sensory modalities may also offer promising non-invasive portals for influencing nervous system function.

## Conclusion

The evolutionary neurophysiological framework of the polyvagal theory provides a plausible model for post-traumatic chronic pain and functional gastrointestinal disorders based on systemic brain-body responses to safety and threat. This framework provides an integrative perspective that unites multiple co-occurring phenomena across perceptual and neurophysiological reports, though much individual variability still remains. While FM and IBS share a neurophysiological substrate with threat-response systems, symptom heterogeneity is the rule rather than the exception, which likely reflects interactions with higher level brain structures, neuroendocrine, and immune processes [e.g., 89)]. However, this review highlights the potential for the autonomic nervous system to be the basis for the organization and synthesis of observations made made by physicians, mental health practitioners, and neuroscientists to piece together the mechanisms that link traumatic experiences, threat-related nervous system function, and multiple somatic disorders. Conversations about trauma history between patients and medical practitioners may be critical for interpreting symptoms and developing treatment plans, but these conversations are rare (26). By building a better understanding of the systemic chronic nervous system alterations that can be induced by trauma, the medical community can move toward explaining co-morbidities and developing targeted treatments.

## Author contributions

JK and SWP both contributed to the conceptualization and writing of this work.

### Conflict of interest statement

The authors declare that the research was conducted in the absence of any commercial or financial relationships that could be construed as a potential conflict of interest. The handling editor is currently co-organizing a Research Topic with one of the authors SWP, and confirms the absence of any other collaboration.

## Abbreviations

DMX: dorsal motor nucleus of the vagus
DVC: dorsal vagal complex
FGID: functional gastrointestinal disorder
FM: fibromyalgia
GI: gastrointestinal
HRV: heart rate variability
IRS: irritable bowel syndrome
NA: nucleus ambiguous
NTS: nucleus of the solitary tract
PTSD: post-traumatic stress disorder
RAVANS: respiratory-gated auricular vagal afferent nerve stimulation
RSA: respiratory sinus arrhythmia
SNS: sympathetic nervous system
t-VNS, transcutaneous electrical vagal nerve stimulation;VVC: ventral vagal complex.
