# Corrigendum: Chronic Diffuse Pain and Functional Gastrointestinal Disorders After Traumatic Stress: Pathophysiology Through a Polyvagal Perspective

**DOI:** 10.3389/fmed.2018.00229

**Published:** 2018-08-15

**Authors:** Jacek Kolacz, Stephen W. Porges

**Affiliations:** ^1^Traumatic Stress Research Consortium, Kinsey Institute, Indiana University, Bloomington, IN, United States; ^2^Department of Psychiatry, University of North Carolina, Chapel Hill, NC, United States

**Keywords:** trauma, polyvagal theory, irritable bowel syndrome, functional gastrointestinal disorders, chronic pain, fibromyalgia, heart rate variability, respiratory sinus arrhythmia

In the published article, there was an error regarding the affiliations for Stephen W. Porges. As well as having affiliation 2, Dr. Stephen W. Porges should also have affiliation 1. The authors apologize for this error and state that this does not change the scientific conclusions of the article in any way.

The original article has been updated.

## Conflict of interest statement

The authors declare that the research was conducted in the absence of any commercial or financial relationships that could be construed as a potential conflict of interest.

